# Medical Societies and Charlatans

**Published:** 1908-02

**Authors:** 


### MEDICAL SOCIETIES AND CHARLATANS.

Crandall has recently* explained the plan and scope of the work carried out by the legal department of the Medical Society of the County of New York and so many important points have been threshed out by this organization that we urge the board of censors in all the societies of the south, and especially in Atlanta, to study carefully this paper with the view of applying the principles that are particularly indicated, in our various cities and towns.

An important duty of the medical societies is to safeguard the financial interests of medical men and the first step in this direction should be the enforcement of the "medical practice" laws, or the enactment of more stringent ones. The up-to-date charlatan, who imitates the latest scientific methods, must be attacked by men of special training and ability. Notwithstanding the many difficulties encountered, some of the strongest and most resolute violators of the medical law of New York have been attacked successfully during the past few years. One of the magistrates of the Court of Special Sessions said not long since, "the Medical Society has practically run out of business the ordinary quack. It now seems to be turning its attention to the quack who is coached by a lawyer, or has worked out some

*Journal American Medical Association, Feb. 8th, 1908.

ingenious method of his own to evade the medical law." If this has been done in New York, why can not we do the same here in Atlanta

A systematic series of prosecutions were taken up for the purpose of establishing legal principles and precedents. Crandall says that the worst offenders were beyond the reach of the usual methods and a systematic widening of lines was necessary. One of the chief difficulties was in obtaining a decision from the highest court of the state defining the "practice of medicine." Finally this was obtained and affirmed by the Supreme Court. In this, Justice Clark insisted that *diagnosis* was an integral and important part of the study and practice of medicine, and that if the definition were confined to mere administration of drugs, or the use of surgical instruments, it would eliminate the very corner stone of successful practice, namely the diagnosis.

The next most important work, Crandall asserts, was the campaign against abortionists. It is said that 100,000 abortions are committed annually in New York. Their counsel, after a series of cases, established the principle that midwives could be convicted for agreeing to perform abortions, or any act tending to consummate the agreement. It was further established that to conduct a house where abortions are habitually performed constitutes a nuisance and as such can be abated.

The quacks who prey upon the credulity of young men, and by extortion and blackmail were found to have procured enormous sums from men driven into mortgaging or pawning the last of their property by these heartless vampires and harpies. Most of these quacks were registered physicians and therefore unusually difficult to reach by ordinary methods; some were successfully prosecuted for grand larceny or false pretense or for false diagnosis, while others were attacked through the Post Office Department, which declared their business fradulent; the newspapers were so notified and advised that to continue the advertisements would mean exclusion of the papers from the mails. Almost immediately, fifty-two vile concerns were exterminated.

